# Hybrid capture-based genomic profiling of circulating tumor DNA from patients with estrogen receptor-positive metastatic breast cancer

**DOI:** 10.1093/annonc/mdx490

**Published:** 2017-08-31

**Authors:** J H Chung, D Pavlick, R Hartmaier, A B Schrock, L Young, B Forcier, P Ye, M K Levin, M Goldberg, H Burris, L M Gay, A D Hoffman, P J Stephens, G M Frampton, D M Lipson, D M Nguyen, S Ganesan, B H Park, L T Vahdat, B Leyland-Jones, T I Mughal, L Pusztai, J O’Shaughnessy, V A Miller, J S Ross, S M Ali

**Affiliations:** 1 Foundation Medicine, Inc., Cambridge;; 2 Avera Cancer Institute, Sioux Falls;; 3 Baylor University Medical Center, Texas Oncology, US Oncology, Dallas;; 4 Sarah Cannon Research Institute, Nashville;; 5 Eastchester Center for Cancer Care, Bronx;; 6 Sutter Medical Group of the Redwoods, Santa Rosa;; 7 Division of Medical Oncology, Department of Medicine, Rutgers Cancer Institute of New Jersey, New Brunswick;; 8 Sidney Kimmel Comprehensive Cancer Center, The Johns Hopkins University School of Medicine, Baltimore;; 9 Weill Cornell Breast Center, Weill Cornell Medicine, New York;; 10 Tufts University Medical Center, Boston;; 11 Department of Breast Medical Oncology, Yale University, Yale Cancer Center, New Haven;; 12 Department of Pathology and Laboratory Medicine, Albany Medical College, Albany, USA

**Keywords:** ctDNA, liquid biopsy, genomic profiling, ER, metastatic breast cancer, ESR1

## Abstract

**Background:**

Genomic changes that occur in breast cancer during the course of disease have been informed by sequencing of primary and metastatic tumor tissue. For patients with relapsed and metastatic disease, evolution of the breast cancer genome highlights the importance of using a recent sample for genomic profiling to guide clinical decision-making. Obtaining a metastatic tissue biopsy can be challenging, and analysis of circulating tumor DNA (ctDNA) from blood may provide a minimally invasive alternative.

**Patients and methods:**

Hybrid capture-based genomic profiling was carried out on ctDNA from 254 female patients with estrogen receptor-positive breast cancer. Peripheral blood samples were submitted by clinicians in the course of routine clinical care between May 2016 and March 2017. Sequencing of 62 genes was carried out to a median unique coverage depth of 7503×. Genomic alterations (GAs) in ctDNA were evaluated and compared with matched tissue samples and genomic datasets of tissue from breast cancer.

**Results:**

At least 1 GA was reported in 78% of samples. Frequently altered genes were *TP53* (38%), *ESR1* (31%) and *PIK3CA* (31%). Temporally matched ctDNA and tissue samples were available for 14 patients; 89% of mutations detected in tissue were also detected in ctDNA. Diverse *ESR1* GAs including mutation, rearrangement and amplification, were observed. Multiple concurrent *ESR1* GAs were observed in 40% of *ESR1-*altered cases, suggesting polyclonal origin; *ESR1* compound mutations were also observed in two cases. *ESR1-*altered cases harbored co-occurring GAs in *PIK3CA* (35%), *FGFR1* (16%), *ERBB2* (8%), *BRCA1/2* (5%), and *AKT1* (4%).

**Conclusions:**

GAs relevant to relapsed/metastatic breast cancer management were identified, including diverse *ESR1* GAs. Genomic profiling of ctDNA demonstrated sensitive detection of mutations found in tissue. Detection of amplifications was associated with ctDNA fraction. Genomic profiling of ctDNA may provide a complementary and possibly alternative approach to tissue-based genomic testing for patients with estrogen receptor-positive metastatic breast cancer.

## Introduction

Genomic changes that characterize primary breast cancer (BC) have been elucidated by extensive genomic profiling studies [1]. Comparative analyses of estrogen receptor-positive (ER+) metastatic BC (mBC) have demonstrated genomic evolution during metastatic progression, and following treatment, such as the enrichment of *HER2* and *ESR1* genomic alterations (GAs) [2, 3]. Clonal evolution can arise due to independent primary lesions, expansion of subclonal populations, or acquisition of novel GAs. Genomic changes following therapy are exemplified by acquired activating *ESR1* GAs that mediate aromatase inhibitor (AI) resistance [2–4].

Clonal evolution processes highlight the importance of profiling a contemporaneous sample that is representative of disease progression to guide further management. However, limitations in performing repeated prospective biopsies of metastatic lesions over the disease course for a patient can present challenges for clinical genomic analysis [5]. Liquid biopsy and sequencing of circulating tumor DNA (ctDNA) from blood could provide a complementary approach to tissue-based genomic testing for mBC.

Research studies of BC identified genomic changes in ctDNA following therapy, however, limited numbers of ER+ BC have been profiled [6–8]. In larger studies of ctDNA from ER+ mBC, droplet digital PCR (ddPCR) identified select mutations in *ESR1* or *PIK3CA* [9–11]. In phase 3 trials for ER+/HER2-negative (HER2−) BC, prospective ctDNA assessment identified patients with *PIK3CA* mutation who derived survival benefit from buparlisib [4]. Retrospective analyses of ctDNA in phase 3 trials suggest that *ESR1* mutations are associated with resistance to AI but not selective ER down-regulators (SERDs), and can guide therapy selection [11].

In this study, we carried out hybrid capture-based genomic profiling to characterize GAs in ctDNA from 254 patients with ER+ BC during the course of their clinical care. 

## Methods

Detailed descriptions of patient samples/methods are presented in supplementary methods, available at *Annals of Oncology* online. Briefly, peripheral blood samples were collected from 254 patients with ER+ BC, plasma was isolated from 20 ml whole blood, ≥20 ng DNA was extracted, and hybrid capture-based genomic profiling of ctDNA was carried out in a CLIA-certified/CAP-accredited laboratory [Foundation Medicine (FM)] to identify substitutions, short insertions/deletions, rearrangements/fusions, and amplifications [12]. Sixty-two genes (supplementary Table S1, available at *Annals of Oncology* online) were sequenced (Illumina HiSeq 2500 or 4000) to a median unique coverage depth of 7503×. Maximum somatic allele frequency (MSAF) was used to estimate the ctDNA fraction in plasma. 

## Results

### Patient characteristics

This study of hybrid capture-based sequencing of ctDNA in blood included consecutive genomic profiling results from 254 female patients with an initial diagnosis of ER+ BC, determined by routine IHC. Patient characteristics are described in Table 1 and supplementary Table S2, available at *Annals of Oncology* online. For patients with available clinical information, 94% were stage IV, 88% had received prior chemotherapy, and 88% had received prior AI in the adjuvant and/or metastatic setting.

**Table 1.** Patient characteristics

		All ER +	ER+/HER2−	ER+/HER2+	ER+/HER2 unk
*N*		254	197	28	29
Median age, years (range)		58 (32–85)	58 (33–85)	57 (33–79)	62 (32–78)
Stage, *N* (%)	I	2 (0.8%)	2 (1.1%)	0 (0%)	0 (0%)
	II	5 (2.1%)	3 (1.6%)	1 (3.7%)	1 (3.8%)
	III	7 (2.9%)	7 (3.7%)	0 (0%)	0 (0%)
	IV	226 (94.2%)	175 (93.6%)	26 (96.3%)	25 (96.2%)
	Unknown	14	10	1	3
Previous chemotherapy^a^	Yes	120 (88.2%)	94 (87.0%)	18 (90.0%)	8 (100%)
	[adj/met/unk], *N*	[24/91/5]	[17/72/5]	[4/14/0]	[3/5/0]
	No	16 (11.8%)	14 (13.0%)	2 (10.0%)	0 (0%)
	Unknown	118	89	8	21
Previous aromatase inhibitor^a^	Yes	115 (88.5%)	95 (92.2%)	15 (75.0%)	5 (71.4%)
	[adj/met/unk], *N*	[19/91/5]	[15/75/5]	[2/13/0]	[2/3/0]
	No	15 (11.5%)	8 (7.8%)	5 (25.0%)	2 (28.6%)
	Unknown	124	94	8	22
Previous tamoxifen^a^	Yes	56 (43.8%)	41 (40.6%)	8 (40.0%)	7 (100%)
	[adj/met/unk], *N*	[41/13/2]	[28/11/2]	[7/1/0]	[6/1/0]
	No	72 (56.2%)	60 (59.4%)	12 (60.0%)	0 (0%)
	Unknown	126	96	8	22
Previous fulvestrant^a^	Yes	69 (54.3%)	57 (56.4%)	7 (36.8%)	5 (71.4%)
	No	58 (45.7%)	44 (43.6%)	12 (63.2%)	2 (28.6%)
	Unknown	127	96	9	22

a See supplementary Table S2, available at *Annals of Oncology* online, for detailed descriptions of treatments in adjuvant/metastatic settings, and for treatment/response status at the time of sample collection.

adj, adjuvant only; met, metastatic or metastatic and adjuvant; unk, unknown.

### GAs identified in ctDNA from ER+ BC

At least 1 GA was detected in 78% of cases with an average of 2.5 GA/sample (range 0–27). MSAF was calculated for each case and provided a median estimated ctDNA fraction of 1.7% (interquartile range 0.3%–9.2%). Eighty-four percent of cases have evidence of ctDNA in the blood (MSAF > 0). There was no evidence of ctDNA in the blood (MSAF = 0) in 15% (33/226) of stage IV cases and 43% (6/14) of stage I–III cases. The most frequently altered genes in ER+ BC were *TP53* (38%), *ESR1* (31%), *PIK3CA* (31%), *CDH1* (10%), and *ERBB2* (8%) (supplementary Figure S1, available at *Annals of Oncology* online).

**Figure 1.**
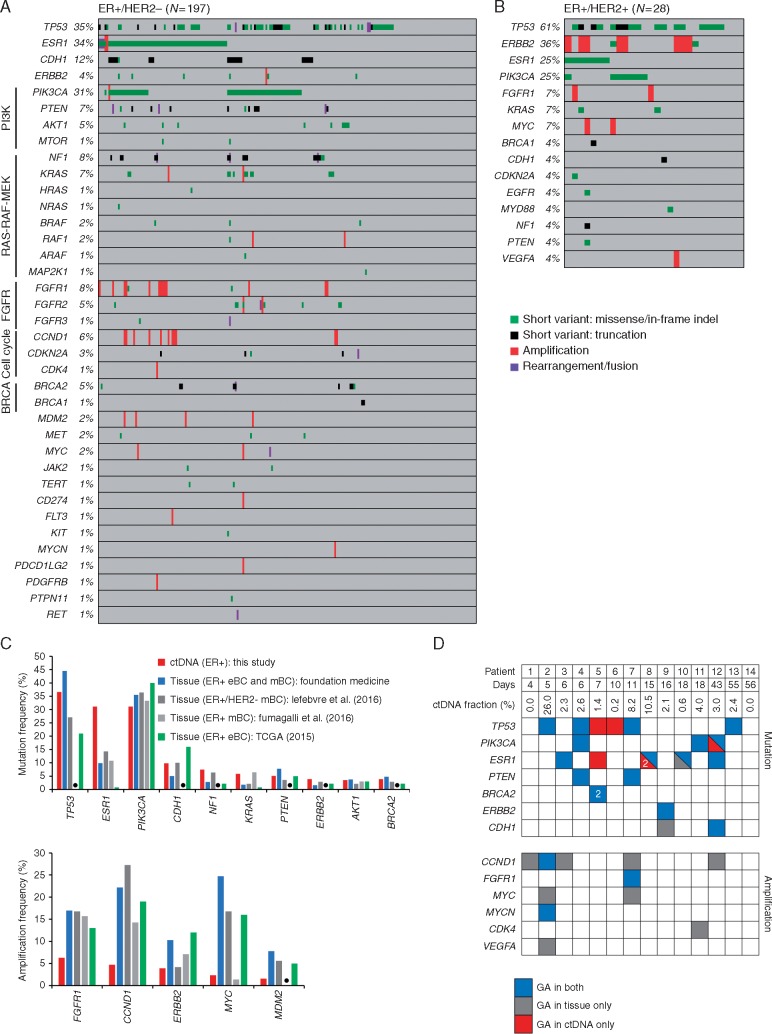
Genomic alterations in ctDNA from patients with ER+ breast cancer and comparisons with tissue. (A) GAs identified in 197 cases of ER+/HER2− BC. Percent of cases altered is indicated. (B) GAs identified in 28 cases of ER+/HER2+ BC. (C) Comparison of the most frequently mutated (top panel) or amplified (bottom panel) genes observed in ctDNA in this study (*N *=* *254) with tissue-based genomic profiling of ER+ BC. Datasets from ER+ BC tissue used for comparison were from the FM database (*N *=* *851) and published studies of tissue from early BC (eBC, TCGA [1]: *N *=* *594) and mBC (Lefebvre et al. [3]: *N *=* *143; Fumagalli et al. [2]: *N *=* *182). Data from [1, 3] were extracted from cBioPortal. Black dots represent genes that were not assessed in [2]. (D) Concordance between GAs found in ctDNA and matched tumor tissue from 14 patients. Days between ctDNA and tissue collection are shown. The ctDNA fraction was estimated using MSAF. Concordant/shared GAs are in blue, GAs found in tissue only are in grey, and GAs found in ctDNA only are in red. For samples with multiple unique mutations in a gene (patient-5 and patient-8), the number of mutations is shown.

For ER+/HER2− BC, the most frequently altered genes were *TP53* (35%)*, ESR1* (34%), *PIK3CA* (31%), and *CDH1* (12%) (Figure 1A). *ERBB2* activating mutations were identified in 3% of cases. *ERBB2* amplification was identified in one patient initially diagnosed with ER+/HER2− BC (IHC on a breast biopsy); gain of HER2 was subsequently confirmed by IHC (3+) on a metastatic biopsy. Activating kinase fusions (*FGFR2-INA*, *FGFR3-TACC3*, *NCOA4-RET*) were observed in three cases (2%). Frequently altered pathways included PI3K-AKT-mTOR (38%), RAS-RAF-MEK (15%), FGFR (14%), cell cycle (8%), and BRCA (6%).

For ER+/HER2+ BC, the most frequently altered genes were *TP53* (61%), *ERBB2* (36%), *PIK3CA* (25%), and *ESR1* (25%) (Figure 1B). *ERBB2* amplification was observed in 29% (8/28) of HER2+ cases, consistent with next-generation sequencing (NGS) studies of ctDNA in HER2+ BC [7, 13]. The estimated ctDNA fraction was significantly higher for HER2+ cases with *ERBB2* amplification compared with cases without *ERBB2* amplification detected (supplementary Figure S2A, available at *Annals of Oncology* online), suggesting that the ability to detect *ERBB2* amplification was associated with the quantity of ctDNA in the blood. 

### Comparison of GAs in ctDNA and tissue

We compared frequently altered genes in ctDNA with ER+ BC tissue samples from the FM database and published studies [1–3] (Figure 1C). For the majority of genes, mutation frequencies in ctDNA were similar to the range observed in tissue; *ESR1* was mutated at a higher frequency (greater than twofold) compared with tissue, as expected from our study population comprising mostly patients who had received or were receiving AI treatment (supplementary Table S2, available at *Annals of Oncology* online) [9]. Amplifications were observed at lower frequencies in ctDNA compared with tissue, consistent with other studies of amplification detection in ctDNA from BC [7, 13]. The estimated ctDNA fraction was significantly higher for cases with an amplification detected compared with cases without (supplementary Figure S2B, available at *Annals of Oncology* online).

Genomic profiles of matched blood and tissue samples collected within 60 days of each other were available for 14 cases. We compared GAs assessed in both ctDNA and tissue (Figure 1D; supplementary Table S3, available at *Annals of Oncology* online). For short variant mutations, 89% (17/19) that were detected in tissue were also detected in ctDNA. Six mutations were detected in ctDNA only and two mutations were in tissue only. One ctDNA only *ESR1* mutation (patient-5) was found in a second tissue sample from a distinct metastatic site, collected 408 days before blood sampling. One case (patient-10) harbored one shared and one tissue only *ESR1* mutation; the allele frequency (AF) for the shared mutation (AF = 34%) was 10-fold higher compared with the tissue only mutation (AF = 3%). Two cases harbored both shared and ctDNA only mutations for the same gene (patient-8: *ESR1*; patient-12: *PIK3CA*): the shared mutation had a higher AF than the ctDNA only mutations in both cases (*ESR1:* twofold; *PIK3CA:* threefold), suggesting that ctDNA only mutations occur in less represented clones that may not be detected in a single tumor biopsy, consistent with clonal heterogeneity. For amplifications, 27% (3/11) that were detected in tissue were also detected in ctDNA; no amplifications were detected in ctDNA only. The estimated ctDNA fraction was higher for two cases where at least one amplification was detected in both tissue and ctDNA than for cases where amplification was detected in tissue only.

### Landscape of *ESR1* alterations in ctDNA

A total of 131 *ESR1* GA were observed in 80 ER+ cases (supplementary Figure S3A, available at *Annals of Oncology* online), including both ER+/HER2− and ER+/HER2+ cases (Figure 1A and B); whereas, only 1 *ESR1* GA (amplification) was observed in the ctDNA of 74 ER- cases (*P = *0.0001, Fisher’s exact test, two-tailed, supplementary Figure S3B, available at *Annals of Oncology* online). For the 130 ER+ patients with available clinical information regarding AI treatment, 35% (40/115) of all AI-treated patients had an *ESR1* GA, and consistent with previous studies [9], *ESR1* GAs were more frequent in patients treated with AI in the metastatic setting (40%, 36/91) versus patients treated with adjuvant AI only (11%, 2/19); all patients (40/40) with *ESR1* GA had received prior AI (supplementary Table S2, available at *Annals of Oncology* online).

**Figure 2.**
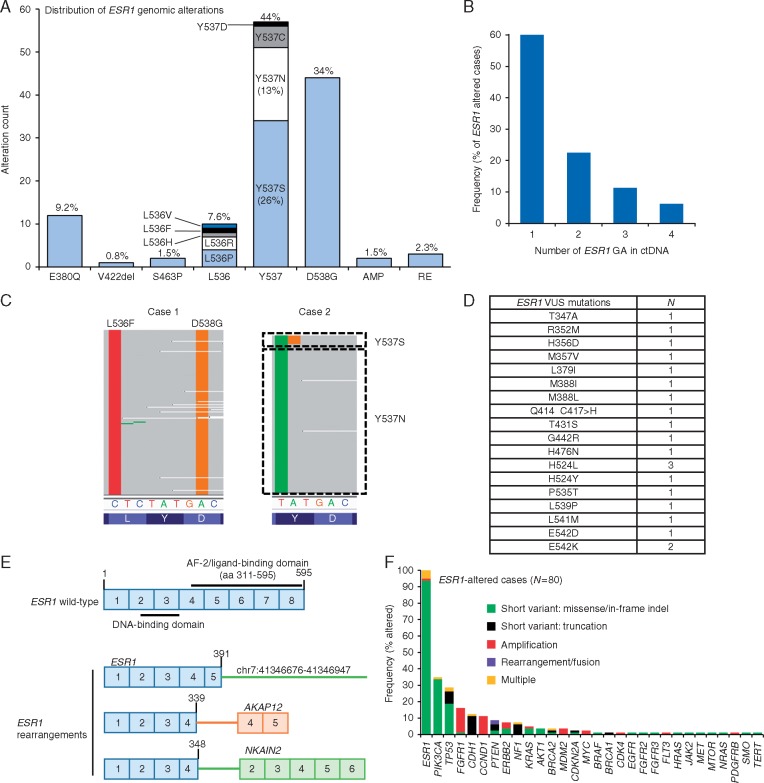
Landscape of *ESR1* alterations in ctDNA. (A) Graph represents the number of cases with each *ESR1* GA. The percent indicate the frequency of each *ESR1* GA relative to all 131 *ESR1* GA identified. AMP, amplification; RE, rearrangement. (B) Percent of cases with 1, 2, 3, or 4 *ESR1* GAs. (C) Compound mutations in *ESR1*: in case-1 all instances of L536F and D538G were observed on the same read in *cis*. In case-2 *ESR1* c.1609T>A (Y537N) was observed alone in the majority of reads, and *ESR1* c.1609T>A and c.1610A>G were seen together as compound mutations in a portion of reads to generate *ESR1* Y537S. (D) *ESR1* variants of unknown significance identified in this study. (E) *ESR1* rearrangements identified in this study. Numbered boxes represent exons and numbers above indicate amino acid position. Rearrangement break points in *ESR1* were in exon 4 or exon 5. (F) Assessment of GAs that co-occur with *ESR1* GA: frequency of *ESR1-*altered cases (*N *=* *80) with alterations in the genes indicated. ‘Multiple’ represents cases harboring multiple classes of genomic alteration.

The most frequent *ESR1* GAs were D538G, Y537S, Y537N, and E380Q. All observed *ESR1* mutations are activating or occur at the L536 position where multiple activating mutations have been characterized (Figure 2A). Of the 80 *ESR1-*altered cases, 40% had >1 *ESR1* GA (range 2–4) (Figure 2B). In comparison, 24% (19/79) of *PIK3CA-*altered cases harbored >1 *PIK3CA* GA (range 2–8) and 23% (22/96) of *TP53*-altered cases harbored >1 *TP53* GA (range 2–11). In cases with >1 *ESR1* mutation, no one *ESR1* mutation had a consistently greater AF than co-occurring *ESR1* mutations, suggesting that diverse *ESR1* mutations could contribute to AI resistance (supplementary Figure S4, available at *Annals of Oncology* online).

Multiple *ESR1* GAs in the same sample are thought to be polyclonal in origin [9, 10]. We carried out a pairwise assessment of all co-occurring *ESR1* mutations to determine whether any mutation pairs existed as compound mutations on the same allele (supplementary Table S4, available at *Annals of Oncology* online). Out of 67 mutation pairs, 49 were close enough to be evaluated on the same sequencing read; compound mutations were observed in 2/49 mutation pairs (Figure 2C). In case-1, *ESR1* L536F/D538G were observed as compound mutations on all reads. In case-2, *ESR1* Y537N occurred as a single mutation in most reads, but a subset of reads harbored a compound mutation at the Y537 codon that resulted in conversion of Y537N to Y537S; the existence of two subsets of reads suggests sequential mutational events.

In addition to the GAs described above, 21 *ESR1* variant of unknown significance (VUS) mutations were identified (Figure 2D) in 15 ER+ cases including 5 cases with no *ESR1* GAs (supplementary Figure S3A, available at *Annals of Oncology* online); no *ESR1* VUS was observed in 74 ER- cases. To evaluate compound mutations, we analyzed 51 co-occurring *ESR1* mutation pairs that involved an *ESR1* VUS, and 20 could be evaluated on the same sequencing read. Compound mutations were observed in 4/20 mutation pairs and 2 mutation pairs existed as compound mutations in only a subset of sequencing reads (supplementary Figure S5, available at *Annals of Oncology* online).

*ESR1* rearrangements were observed in three cases: two *ESR1* rearrangements had potential 3’ fusion partners (*AKAP12*, *NKAIN2*) and one *ESR1* rearrangement was fused to an intergenic region (Figure 2E). *ESR1-AKAP12* is recurrent in BC and all three *ESR1* rearrangements resulted in loss of the ligand-binding domain (LBD), which likely results in constitutive ER activation [14, 15]. Each *ESR1-*rearranged case harbored concurrent *ESR1* mutation, suggesting prior AI exposure: we confirmed prior adjuvant AI and fulvestrant treatment of the patient with *ESR1* fused to intergenic space.

### Co-occurring GAs with *ESR1*

To inform therapeutic strategies for AI refractory patients, we evaluated co-occurring alterations with *ESR1* GAs and identified concurrent GAs that have been associated with responses to targeted therapy in BC [4] including *PIK3CA* (35%), *FGFR1* (16%), *ERBB2* (8%), *BRCA1/2* (5%), and *AKT1* (4%) (Figure 2F).

For cases with concurrent *PIK3CA*/*ESR1* mutation, the *PIK3CA*:*ESR1* AF ratio was ≥1 for 75% (21/28) of cases, consistent with *PIK3CA* being a truncal driver and *ESR1* arising following AI (supplementary Figure S6, available at *Annals of Oncology* online). Concurrent *ESR1/ERBB2* mutation was more frequent in ctDNA than tissue: in ctDNA, 4% (3/79) of *ESR1-*mutated cases had concurrent *ERBB2* mutation; whereas, in the FM database, 0.6% (6/969) of *ESR1*-mutated BC tissue samples had concurrent *ERBB2* mutation.

## Discussion

Genomic profiling of ctDNA has the potential to capture GAs that drive recurrent disease or therapeutic resistance and may provide an alternative when tissue biopsy is challenging. However, genomic profiles of ctDNA from ER+ mBC have not been extensively studied. We describe GAs identified in ctDNA from the blood of 254 patients with ER+ mBC.

Eighty-four percent of samples had evidence of ctDNA in the blood, consistent with a study of ctDNA release in mBC [8]. For cases with no evidence of ctDNA in the blood, lack of detectable somatic alterations is, in part, likely associated with insufficient ctDNA release into the blood at the time point of sampling that can be affected by clinical parameters including disease stage, tumor size, number of metastatic sites, albumin level, and number of lines of treatment [8, 16]; these parameters were variable in this study of unselected cases (supplementary Table S2, available at *Annals of Oncology* online).

Alterations were identified in genes that have been associated with responses to targeted therapy in ER+ BC (*PIK3CA*, *ESR1, ERBB2*, *FGFR1*, *BRCA1/2*, *AKT1*) [4]. Compared with genomic studies of ER+ BC tissue biopsies, we identified similar mutation frequencies in ctDNA [1–3]. Tumor burden can be monitored by longitudinal assessment of variant AFs in ctDNA [8]. However, genomic profiling of large numbers of genes is best-suited for guiding therapy selection, but may be cost-prohibitive for serial testing. Instead, genomic profiling could establish GAs present in ctDNA at baseline for a patient, and guide design of personalized serial monitoring assays. GAs reported here could inform prioritization of genes to include for limited sequencing panels for longitudinal disease monitoring of ER+ mBC.

For a smaller subset of patients with temporally matched ctDNA and tissue samples, 89% of short variant mutations that were detected in tissue were also detected in ctDNA. Additional *ESR1*, *TP53*, and *PIK3CA* mutations were identified only in ctDNA; other studies have similarly observed additional mutations for each of these genes in ctDNA compared with matched tissue [5, 10, 17]. Additional mutations in ctDNA could reflect the utility of liquid biopsy to capture heterogeneity of metastatic sites in ER+ mBC [6]. Consistent with this idea, for paired cases with both shared and ctDNA only mutations in one gene, the shared mutation AF was two to threefold higher than the ctDNA only mutation AF. This hypothesis warrants confirmation in prospective trials and may be more relevant in clinical settings where targeted therapies are routinely employed.

In this study, genomic profiling was carried out as part of routine clinical care for unselected patients, including patients with low tumor burden; therefore, many samples had a low estimated ctDNA fraction (supplementary Table S2, available at *Annals of Oncology* online). The sensitivity for amplification detection in ctDNA was 27% for the 14 matched ctDNA-tissue pairs. Amplifications (including *CCND1, FGFR1, ERBB2*) were detected in ctDNA at lower frequencies than tissue; specifically *ERBB2* amplification was identified in 29% of HER2+ cases. Detection of amplifications was associated with higher estimated ctDNA fraction. Our findings are consistent with NGS studies [using NGS panels or alternative approaches for amplification detection such as low coverage whole genome sequencing (plasma-Seq)] that highlighted the limitations for robust detection of amplifications in the context of low ctDNA fractions [18, 19]. Other studies identified similarly low frequencies of *ERBB2* amplification (21%–32%) in ctDNA from HER2+ BC and detected other common BC amplifications (including *CCND1, FGFR1*) at significantly lower frequencies in ctDNA compared with matched tissue [7, 13]. Therefore, although amplifications may be detected in a subset of cases with sufficient ctDNA fraction, tissue-based genomic testing may be a more reliable method of detection. In BC, *ERBB2* amplification remains the only established clinically utilized copy number biomarker, but amplifications including *FGFR1* and *11q13* are being evaluated as biomarkers in trials [4]; tissue-based testing may be the preferable method for treatment selection based on copy number biomarkers.

We observed a high frequency of *ESR1* GAs that are associated with AI resistance, as expected for this patient population of mostly mBC with prior AI treatment [9]. The *ESR1* mutation frequency reported here is consistent with studies of AI-treated, ER+ mBC that used ddPCR to assess selected *ESR1* mutations in ctDNA, including frequencies reported in several phase 3 trials [4, 9–11].

We observed a similar distribution of *ESR1* mutations compared with a study of common *ESR1* mutations in ctDNA using ddPCR [10]. Consistent with other studies, we frequently observed cases harboring >1 *ESR1* GA [9, 10]. Multiple *ESR1* mutations are thought to reflect convergent evolution of distinct clones during AI resistance [6, 10]; for the few cases evaluated using dual mutation-specific ddPCR probes, different *ESR1* mutations existed on separate alleles [17, 20]. We confirmed that most *ESR1* mutation pairs occur on distinct sequencing reads, likely reflecting polyclonal origin; however, we also identified cases with *ESR1* compound mutations on the same allele. Studies of *ESR1* have focused on single mutations; *ESR1* compound mutations in *cis* warrant further study, and such mutations might display differential therapeutic sensitivities compared with characterized single mutations.

Diverse *ESR1* alterations were observed, including rearrangements with break points resulting in loss of the LBD. Similar *ESR1* rearrangements with variable 3’ fusion partners have been described and are activating [14, 15]. *ESR1* rearrangements demonstrate preclinical resistance to AI and SERDs, therefore, detection of *ESR1* rearrangements may be important for therapy selection [15]. *ESR1* VUSs reported here could represent novel functional mutations that warrant characterization.

We identify co-occurring alterations with *ESR1*, which could represent alternative targets or rational targets for combination therapy with SERDs [4]*.* Some of these GAs have been successfully targeted in the context of co-occurring *ESR1* GA: responses have been observed for patients with concurrent *ESR1/AKT1* mutation (to AZD5363) [21], *ESR1/PIK3CA* mutation (to alpelisib) [22], and for a patient from this study with *ESR1/BRCA2* mutation (to olaparib; Dr S. Blau, personal communication).

Concurrent *ESR1/ERBB2* activating mutations occurred in ER+/HER2− BC and were more frequently observed in ctDNA compared with tissue, suggesting that *ERBB2* and *ESR1* mutations may commonly reside on distinct clones that may not be detected in a single tissue biopsy; *ESR1* mutations were also observed in 25% of ER+/HER2+ BC. Combinations of SERDs with HER2-targeted therapy could be relevant for such cases.

Here, we demonstrate the clinical implementation of genomic profiling of ctDNA from patients with ER+ BC and identify clinically relevant GAs. Blood-based testing may provide an alternative or complementary approach to tissue-based genomic testing for patients with mBC.

## Supplementary Material

mdx490_supplementary_figure_s1

mdx490_supplementary_fig_s2

mdx490_supplementary_figure_s3-s6

mdx490_supplementary_table_s1_s3_s4

mdx490_supplementary_table_s2

mdx490_supplementary_methods_corrected
