# Supplementary material for: Hybrid capture-based genomic profiling of circulating tumor DNA from patients with estrogen receptor-positive metastatic breast cancer
Source: Ann Oncol. 2017 Aug 31;28(11):2866–73. doi: 10.1093/annonc/mdx490 (PMC5834148; doi:10.1093/annonc/mdx490)
Supplement: mdx490_supplementary_figure_s1 [file mdx490_supplementary_figure_s1.docx]

**Supplementary Figure S1**

Frequency of GAs (% cases with GA) in **(a)** all ER+ cases, **(b)** all ER+/HER2- cases, **(c)** all ER+/HER2+ cases **(d)** stage IV ER+ cases **(e)** stage IV ER+/HER2- cases, and **(f)** stage IV ER+/HER2+ cases. “Multiple” represents cases harboring multiple classes of genomic alteration.
