# Supplementary material for: Hybrid capture-based genomic profiling of circulating tumor DNA from patients with estrogen receptor-positive metastatic breast cancer
Source: Ann Oncol. 2017 Aug 31;28(11):2866–73. doi: 10.1093/annonc/mdx490 (PMC5834148; doi:10.1093/annonc/mdx490)
Supplement: mdx490_supplementary_fig_s2 [file mdx490_supplementary_fig_s2.docx]

**Supplementary Figure S2**

Detection of copy number amplification is associated with increased ctDNA fraction: **(a)** ctDNA fraction estimate (MSAF) for 28 ER+/HER2+ cases with (red) or without (grey) *ERBB2* amplification detected in ctDNA. MSAF was significantly higher for cases with an *ERBB2* amplification (*P*=0.001, t-test, two-tailed). **(b)** MSAF ctDNA fraction estimate for all 254 ER+ cases with (red) or without (grey) at least 1 amplification detected in ctDNA. MSAF was significantly higher for cases with an amplification (*P*<0.001, t-test, two-tailed). Box-and-whisker plots: box spans first and third quartiles, the median is denoted by the horizontal line in the box, whiskers indicate maximum and minimum values within 1.5X the inter-quartile range.
