# Supplementary material for: Hybrid capture-based genomic profiling of circulating tumor DNA from patients with estrogen receptor-positive metastatic breast cancer
Source: Ann Oncol. 2017 Aug 31;28(11):2866–73. doi: 10.1093/annonc/mdx490 (PMC5834148; doi:10.1093/annonc/mdx490)
Supplement: mdx490_supplementary_figure_s3-s6 [file mdx490_supplementary_figure_s3-s6.docx]

**Supplementary Figure S3**

**(a)** Landscape of *ESR1* GA and VUS identified in ER+ BC. **(b)** 74 ER-negative cases were evaluated for comparison of *ESR1* GA with ER+ cases.

**Supplementary Figure S4**

For each case with >1 *ESR1* mutation the relative AF of each *ESR1* mutation was compared to the allele fraction of the most represented *ESR1* mutation in that sample. Box: bottom line = 1^st^ quartile; middle line = median; top line = 3^rd^ quartile. Whiskers represent maximum or minimum values within 1.5 times the interquartile range.


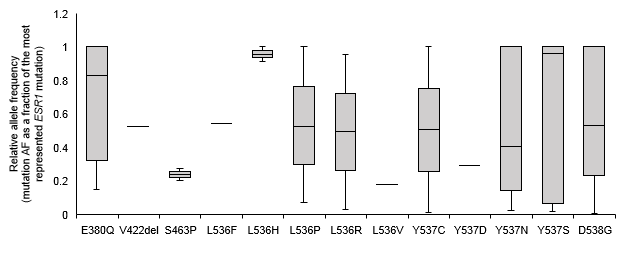


**Supplementary Figure S5**

Compound mutations of *ESR1* GAs and *ESR1* VUS mutations.

**Supplementary Figure S6**

For each of the 28 cases that had co-occurring *PIK3CA* and *ESR1* mutation, relative allele frequency of *PIK3CA/ESR1* was calculated by taking the ratio of the most represented *PIK3CA* mutation and the most represented *ESR1* mutation.
