# Supplementary material for: Hybrid capture-based genomic profiling of circulating tumor DNA from patients with estrogen receptor-positive metastatic breast cancer
Source: Ann Oncol. 2017 Aug 31;28(11):2866–73. doi: 10.1093/annonc/mdx490 (PMC5834148; doi:10.1093/annonc/mdx490)
Supplement: mdx490_supplementary_table_s1_s3_s4 [file mdx490_supplementary_table_s1_s3_s4.docx]

**Supplementary Table S1:** List of 62 genes sequenced in this study using the FoundationACT ctDNA assay.

*Genes that were evaluable for copy number amplification

| *ABL1** |
| --- |
| *AKT1* |
| *ALK** |
| *ARAF** |
| *BRAF** |
| *BRCA1* |
| *BRCA2* |
| *BTK* |
| *CCND1** |
| *CD274 (PD-L1)** |
| *CDH1* |
| *CDK4** |
| *CDK6** |
| *CDKN2A* |
| *CRKL** |
| *CTNNB1* |
| *DDR2** |
| *EGFR** |
| *ERBB2** |
| *ERRFI1* |
| *ESR1** |
| *EZH2* |
| *FGFR1** |
| *FGFR2** |
| *FGFR3** |
| *FLT3** |
| *FOXL2* |
| *GNA11* |
| *GNAQ* |
| *GNAS* |
| *HRAS* |
| *IDH1* |
| *IDH2* |
| *JAK2* |
| *JAK3* |
| *KIT** |
| *KRAS** |
| *MAP2K1* |
| *MAP2K2** |
| *MDM2** |
| *MET** |
| *MPL* |
| *MTOR** |
| *MYC** |
| *MYCN** |
| *MYD88* |
| *NF1* |
| *NPM1* |
| *NRAS* |
| *PDCD1LG2 (PD-L2)** |
| *PDGFRA* |
| *PDGFRB** |
| *PIK3CA** |
| *PTEN* |
| *PTPN11* |
| *RAF1** |
| *RET** |
| *ROS1* |
| *SMO** |
| *TERT-promoter* |
| *TP53* |
| *VEGFA** |

**Supplementary Table S3:** Details of GAs in the 14 paired ctDNA/tissue samples assessed in Figure 1d. Only GAs assessed by both assays were included in the analysis.

| **Patient** | **Shared GA** | **ctDNA only GA** | **Tumor DNA only GA** |
| --- | --- | --- | --- |
| 1 |  |  | *CCND1:* amplification |
| 2 | *CCND1:* amplification *MYCN:* amplification *TP53:* S214F |  | *MYC:* amplification *VEGFA:* amplification |
| 3 | *ESR1:* D538G |  | *CCND1:* amplification |
| 4 | *PIK3CA:* H1047L *PTEN:* splice site 1026+1G>T *TP53:* R213* |  |  |
| 5 | *BRCA2:* V1283fs*2 *BRCA2:* D1280_N1288del | *ESR1:* Y537N *TP53:* R273H |  |
| 6 |  | *TP53:* C277G |  |
| 7 | *FGFR1:* amplification *PTEN:* P103fs*3 *TP53:* C135F |  | *CCND1:* amplification *MYC:* amplification |
| 8 | *ESR1:* Y537S  (ctDNA AF=10.5%) | *ESR1:* L536F  (ctDNA AF=5.7%)  *ESR1:* D538G  (ctDNA AF=5.7%) |  |
| 9 | *ERBB2:* A775_776insYVMA |  | *CDH1:* L249fs*1 |
| 10 | *ESR1:* Y537C  (tissue AF=34%) |  | *ESR1:* L536R  (tissue AF=3%) |
| 11 | *PIK3CA:* H1047R |  | *CDK4:* amplification |
| 12 | *CDH1:* splice site 1565+1G>T *ESR1:* E380Q *PIK3CA:* E545K  (ctDNA AF=3.0%) | *PIK3CA:* E726K  (ctDNA AF=0.9%) | *CCND1:* amplification |
| 13 | *TP53:* R175H |  |  |
| 14 |  |  |  |

**Supplementary Table S4:** Pairwise analysis of co-occurring *ESR1* short variant mutation pairs to determine if mutations occur on the same sequencing read (CIS) or on separate sequencing reads (TRANS). “BOTH” indicates that both CIS and TRANS were observed. “AMBIGUOUS” indicates the mutation pairs could not be assessed on the same sequencing read due to distance between mutations. VUS mutations are in red.

| **Sample** | ***ESR1* mutation 1** | ***ESR1* mutation 2** | **Status of *ESR1* pair** |
| --- | --- | --- | --- |
| 1 | L536P | D538G | TRANS |
| 2 | L379I | Y537N | AMBIGUOUS |
|  | L379I | Y537S | AMBIGUOUS |
|  | L379I | D538G | AMBIGUOUS |
|  | Y537N | Y537S | TRANS |
|  | Y537N | D538G | TRANS |
|  | Y537S | D538G | TRANS |
| 3 | L536F | Y537S | TRANS |
|  | L536F | D538G | CIS |
|  | Y537S | D538G | TRANS |
| 4 | Y537S | D538G | TRANS |
| 5 | Y537N | Y537S | TRANS |
|  | Y537N | D538G | TRANS |
|  | Y537S | D538G | TRANS |
| 6 | Y537N | Y537S | TRANS |
| 7 | E380Q | M388L | TRANS |
|  | E380Q | H476N | AMBIGUOUS |
|  | E380Q | H524Y | AMBIGUOUS |
|  | E380Q | Y537N | AMBIGUOUS |
|  | E380Q | Y537S | AMBIGUOUS |
|  | E380Q | D538G | AMBIGUOUS |
|  | M388L | H476N | AMBIGUOUS |
|  | M388L | H524Y | AMBIGUOUS |
|  | M388L | Y537N | AMBIGUOUS |
|  | M388L | Y537S | AMBIGUOUS |
|  | M388L | D538G | AMBIGUOUS |
|  | H476N | H524Y | AMBIGUOUS |
|  | H476N | Y537N | AMBIGUOUS |
|  | H476N | Y537S | AMBIGUOUS |
|  | H476N | D538G | AMBIGUOUS |
| **Sample** | ***ESR1* mutation 1** | ***ESR1* mutation 2** | **Status of *ESR1* pair** |
| 7 | H524Y | Y537N | BOTH |
|  | H524Y | Y537S | TRANS |
|  | H524Y | D538G | TRANS |
|  | Y537N | Y537S | BOTH |
|  | Y537N | D538G | TRANS |
|  | Y537S | D538G | TRANS |
| 8 | E380Q | Y537N | AMBIGUOUS |
|  | E380Q | D538G | AMBIGUOUS |
|  | Y537N | D538G | TRANS |
|  | L536R | E380Q | AMBIGUOUS |
|  | L536R | Y537N | TRANS |
|  | L536R | D538G | TRANS |
| 9 | L536R | Y537N | TRANS |
|  | L536R | D538G | TRANS |
|  | L536R | E542K | TRANS |
|  | Y537N | D538G | TRANS |
|  | Y537N | E542K | TRANS |
|  | D538G | E542K | TRANS |
| 10 | Y537S | D538G | TRANS |
| 11 | Y537S | D538G | TRANS |
| 12 | Y537N | D538G | TRANS |
| 13 | Y537N | D538G | TRANS |
| 14 | Y537N | Y537S | TRANS |
| 15 | L536H | Y537N | TRANS |
|  | L536H | D538G | TRANS |
|  | L536H | L541M | BOTH |
|  | Y537N | D538G | TRANS |
|  | Y537N | L541M | TRANS |
|  | D538G | L541M | TRANS |
| 16 | Y537S | L536V | TRANS |
|  | Y537S | L536P | TRANS |
|  | Y537S | E542D | TRANS |
|  | L536V | L536P | TRANS |
|  | L536V | E542D | TRANS |
|  | L536P | E542D | TRANS |
| 17 | Q414_C417>H | D538G | AMBIGUOUS |
| 18 | Y537D | Y537C | TRANS |
|  | Y537D | D538G | TRANS |
|  | Y537C | D538G | TRANS |
| 19 | Y537N | Y537S | TRANS |
| 20 | E380Q | Y537S | AMBIGUOUS |
|  | E380Q | D538G | AMBIGUOUS |
| **Sample** | ***ESR1* mutation 1** | ***ESR1* mutation 2** | **Status of *ESR1* pair** |
| 20 | Y537S | D538G | TRANS |
| 21 | V422del | Y537S | AMBIGUOUS |
|  | V422del | D538G | AMBIGUOUS |
|  | Y537S | D538G | TRANS |
| 22 | Y537N | Y537S | TRANS |
|  | Y537N | D538G | TRANS |
|  | Y537S | D538G | TRANS |
| 23 | S463P | L536P | AMBIGUOUS |
|  | S463P | D538G | AMBIGUOUS |
|  | L536P | D538G | TRANS |
| 24 | Y537S | D538G | TRANS |
| 25 | P535T | Y537S | CIS |
| 26 | H356D | D538G | AMBIGUOUS |
|  | H356D | L539P | AMBIGUOUS |
|  | H356D | E542K | AMBIGUOUS |
|  | D538G | L539P | TRANS |
|  | D538G | E542K | TRANS |
|  | L539P | E542K | TRANS |
| 27 | Y537S | D538G | TRANS |
| 28 | Y537N | D538G | TRANS |
| 29 | Y537N | Y537S | TRANS |
| 30 | Y537S | D538G | TRANS |
| 31 | T347A | E380Q | AMBIGUOUS |
|  | T347A | M388I | AMBIGUOUS |
|  | T347A | S463P | AMBIGUOUS |
|  | T347A | H524L | AMBIGUOUS |
|  | T347A | Y537C | AMBIGUOUS |
|  | T347A | D538G | AMBIGUOUS |
|  | E380Q | M388I | CIS |
|  | E380Q | S463P | AMBIGUOUS |
|  | E380Q | H524L | AMBIGUOUS |
|  | E380Q | Y537C | AMBIGUOUS |
|  | E380Q | D538G | AMBIGUOUS |
|  | M388I | S463P | AMBIGUOUS |
|  | M388I | H524L | AMBIGUOUS |
|  | M388I | Y537C | AMBIGUOUS |
|  | M388I | D538G | AMBIGUOUS |
|  | S463P | H524L | AMBIGUOUS |
|  | S463P | Y537C | AMBIGUOUS |
|  | S463P | D538G | AMBIGUOUS |
|  | H524L | Y537C | TRANS |
|  | H524L | D538G | TRANS |
| **Sample** | ***ESR1* mutation 1** | ***ESR1* mutation 2** | **Status of *ESR1* pair** |
| 31 | Y537C | D538G | TRANS |
| 32 | L536R | D538G | TRANS |
| 33 | E380Q | D538G | AMBIGUOUS |
| 34 | E380Q | G442R | AMBIGUOUS |
