# Supplementary material for: Hybrid capture-based genomic profiling of circulating tumor DNA from patients with estrogen receptor-positive metastatic breast cancer
Source: Ann Oncol. 2017 Aug 31;28(11):2866–73. doi: 10.1093/annonc/mdx490 (PMC5834148; doi:10.1093/annonc/mdx490)
Supplement: mdx490_supplementary_table_s2 [file mdx490_supplementary_table_s2.docx]

**Supplementary Table S2: Detailed clinical annotation of treatments and status of treatment/response at the time of sample collection.**

|  |  |  | **Adjuvant/Neoadjuvant** | | | **Metastatic** | | | | **Status at blood collection** | |  |  |
| --- | --- | --- | --- | --- | --- | --- | --- | --- | --- | --- | --- | --- | --- |
| **Case** | **ESR1 alteration** | **ESR1 type** | **Chemotherapy** | **Tamoxifen** | **Aromatase inhibitor** | **Chemotherapy** | **Tamoxifen** | **Aromatase inhibitor** | **Fulvestrant** | **Treatment** | **Response status** | **Biomarker** | **MSAF (%)** |
| 1 | T431S | VUS | No | No | Yes - anastrazole | No | No | Yes - exemestane | Yes | exemestane/everolimus/casodex | progression at last scan | ER+/HER2- | 0.19 |
| 2 | Y537S | known/likely GA | Yes | No | No | Yes | No | Yes - letrozole, exemestane | Yes | unknown | response unknown but evidence of metastatic disease | ER+/HER2+ | 66.24 |
| 3 | Y537S | known/likely GA | Metastatic at diagnosis | | | Yes | Yes | Yes - letrozole, exemestane | Yes | eribulin | stable at last scan | ER+/HER2- | 0.83 |
| 4 | H524L | VUS | Yes | Yes | Yes - letrozole | Yes | No | Yes - letrozole | Yes | unknown | response unknown but evidence of metastatic disease | ER+/unk | 2.36 |
| 5 | L536P | known/likely GA | Metastatic at diagnosis | | | No | No | Yes - anastrazole | Yes | fulvestrant | Partial response | ER+/HER2- | 1.12 |
|  | D538G | known/likely GA |  |  |  |  |  |  |  |  |  |  |  |
| 6 | Y537N | known/likely GA | Unknown | | | | | | | | | ER+/HER2- | 11.92 |
|  | D538G | known/likely GA |  |  |  |  |  |  |  |  |  |  |  |
|  | Y537S | known/likely GA |  |  |  |  |  |  |  |  |  |  |  |
|  | L379I | VUS |  |  |  |  |  |  |  |  |  |  |  |
| 7 | D538G | known/likely GA | Metastatic at diagnosis | | | Yes | No | Yes - letrozole | Yes | Letrozole | progression | ER+/HER2- | 0.87 |
| 8 | E380Q | known/likely GA | Unknown | | | | | | | | | ER+/HER2- | 3.83 |
| 9 | M357V | VUS | Unknown | | | | | | | | | ER+/HER2- | 0.52 |
| 10 | Y537C | known/likely GA | Unknown | | | | | | | | | ER+/HER2- | 16.58 |
| 11 | Y537N | known/likely GA | Unknown | | | | | | | | | ER+/unk | 20.8 |
| 12 | E380Q | known/likely GA | Unknown | | | | | | | | | ER+/unk | 1.41 |
| 13 | L536F | known/likely GA | No | No | Yes - anastrazole | Yes | No | Yes - exemestane | Yes | Gemcitabine | response unknown but evidence of metastatic disease | ER+/HER2- | 10.54 |
|  | D538G | known/likely GA |  |  |  |  |  |  |  |  |  |  |  |
|  | Y537S | known/likely GA |  |  |  |  |  |  |  |  |  |  |  |
| 14 | D538G | known/likely GA | Unknown | | | | | | | | | ER+/HER2- | 23.91 |
| 15 | E380Q | known/likely GA | Unknown | | | | | | | | | ER+/HER2- | 8.03 |
| 16 | Y537S | known/likely GA | Unknown | | | | | | | | | ER+/unk | 3.34 |
| 17 | Y537S | known/likely GA | Unknown | | | Yes | No | Yes - letrozole, exemestane | Yes | fulvestrant/palbociclib (on hold at time of sample due to GI infection) | Stable bone metastases at last scan | ER+/HER2- | 19.81 |
| 18 | Y537S | known/likely GA | No | Yes | No | Yes | No | Yes - letrozole | Yes | letrozole/palbociclib | Progression | ER+/HER2- | 3.45 |
|  | D538G | known/likely GA |  |  |  |  |  |  |  |  |  |  |  |
| 19 | Y537S | known/likely GA | Unknown | | | | | | | | | ER+/HER2- | 5.83 |
| 20 | D538G | known/likely GA | No | Yes | No | Yes | No | Yes - anastrazole, letrozole | Yes | doxorubicin | Progression | ER+/HER2- | 15.26 |
|  | Y537S | known/likely GA |  |  |  |  |  |  |  |  |  |  |  |
|  | Y537N | known/likely GA |  |  |  |  |  |  |  |  |  |  |  |
|  | Y537S | known/likely GA |  |  |  |  |  |  |  |  |  |  |  |
| 21 | D538G | known/likely GA | Metastatic at diagnosis | | | Yes | No | Yes - anastrazole, exemestane | Yes | capecitabine | stable on PET, decrease in CA15-3 | ER+/HER2- | 0.18 |
| 22 | Y537N | known/likely GA | Unknown | | | | | | | | | ER+/HER2+ | 4.93 |
|  | Y537S | known/likely GA |  |  |  |  |  |  |  |  |  |  |  |
| 23 | E380Q | known/likely GA | Unknown | | | | | | | | | ER+/HER2- | 14.15 |
|  | Y537S | known/likely GA |  |  |  |  |  |  |  |  |  |  |  |
|  | Y537N | known/likely GA |  |  |  |  |  |  |  |  |  |  |  |
|  | D538G | known/likely GA |  |  |  |  |  |  |  |  |  |  |  |
|  | M388L | VUS |  |  |  |  |  |  |  |  |  |  |  |
|  | H476N | VUS |  |  |  |  |  |  |  |  |  |  |  |
|  | H524Y | VUS |  |  |  |  |  |  |  |  |  |  |  |
| 24 | E380Q | known/likely GA | received aromatase inhibitor, fulvestrant, palbociclib - unknown if in adjuvant/neoadjuvant or metastatic setting | | | | | | | | | ER+/HER2- | 0.37 |
| 25 | E380Q | known/likely GA | Yes | No | No | Yes | No | Yes - letrozole, exemestane | Yes | ixempra/xeloda/herceptin | stable disease | ER+/HER2+ | 29.69 |
|  | Y537N | known/likely GA |  |  |  |  |  |  |  |  |  |  |  |
|  | D538G | known/likely GA |  |  |  |  |  |  |  |  |  |  |  |
|  | L536R | known/likely GA |  |  |  |  |  |  |  |  |  |  |  |
| 26 | L536R | known/likely GA | Unknown | | | | | | | | | ER+/unk | 13.91 |
|  | E542K | VUS |  |  |  |  |  |  |  |  |  |  |  |
|  | Y537N | known/likely GA |  |  |  |  |  |  |  |  |  |  |  |
|  | D538G | known/likely GA |  |  |  |  |  |  |  |  |  |  |  |
| 27 | D538G | known/likely GA | Unknown | | | | | | | | | ER+/HER2- | 36.87 |
| 28 | Y537N | known/likely GA | Metastatic at diagnosis | | | Yes | Yes | Yes - letrozole | No | capecitabine (discontiuned for 2 weeks at time of sample) | Progression | ER+/HER2- | 6.11 |
| 29 | D538G | known/likely GA | received chemotherapy - not stated if in adjuvant/neoadjuvant or metastatic setting; hormonal therapy - unknown | | | | | | | | | ER+/HER2- | 31.58 |
|  | Y537S | known/likely GA |  |  |  |  |  |  |  |  |  |  |  |
| 30 | Y537S | known/likely GA | Unknown | | | | | | | | | ER+/HER2- | 1.88 |
|  | D538G | known/likely GA |  |  |  |  |  |  |  |  |  |  |  |
| 31 | Y537C | known/likely GA | Unknown | | | | | | | | | ER+/HER2- | 0.64 |
| 32 | Y537N | known/likely GA | Yes | Yes | No | Yes | No | Yes - anastrazole, exemestane, letrozole | Yes | taxol/carboplatin/olaparib | Progression | ER+/HER2- | 1.36 |
| 33 | D538G | known/likely GA | received chemotherapy in metastatic setting; hormonal therapy - unknown | | | | | | | taxol/carboplatin | unknown | ER+/HER2- | 0.55 |
|  | Y537N | known/likely GA |  |  |  |  |  |  |  |  |  |  |  |
| 34 | D538G | known/likely GA | Yes | No | No | Yes | No | Yes - letrozole, exemestane, anastrazole | Yes | CMF | stable disease | ER+/HER2- | 42.37 |
|  | Y537N | known/likely GA |  |  |  |  |  |  |  |  |  |  |  |
| 35 | D538G | known/likely GA | Yes | No | Yes - letrozole | Yes | No | Yes - anastrazole | No | anastrazole | Progression | ER+/HER2- | 12.5 |
| 36 | Y537S | known/likely GA | Unknown | | | Yes | Unknown | | | eribulin | stable | ER+/HER2- | 3.1 |
|  | Y537N | known/likely GA |  |  |  |  |  |  |  |  |  |  |  |
| 37 | Y537S | known/likely GA | Unknown | | | | | | | | | ER+/HER2- | 3.33 |
| 38 | Y537S | known/likely GA | Unknown | | | | | | | | | ER+/HER2- | 14.3 |
| 39 | Y537N | known/likely GA | Unknown | | | | | | | | | ER+/HER2- | 30.6 |
|  | D538G | known/likely GA |  |  |  |  |  |  |  |  |  |  |  |
|  | L536H | known/likely GA |  |  |  |  |  |  |  |  |  |  |  |
|  | L541M | VUS |  |  |  |  |  |  |  |  |  |  |  |
| 40 | D538G | known/likely GA | Unknown | | | | | | | | | ER+/HER2- | 34.59 |
| 41 | D538G | known/likely GA | Yes | Yes | Yes - anastrazole | Yes | No | Yes - exemestane, letrozole | No | letrozole/palbociclib | Progression | ER+/HER2- | 27.67 |
| 42 | Y537S | known/likely GA | Metastatic at diagnosis | | | Yes | No | Yes - anastrazole | No | carboplatin/taxol/avastin | response at last scan | ER+/HER2- | 24.13 |
| 43 | ESR1 rearrangement | known/likely GA | Yes | No | Yes | Yes | No | No | Yes | capecitabine | progression | ER+/HER2- | 2.85 |
|  | E542D | VUS |  |  |  |  |  |  |  |  |  |  |  |
|  | L536P | known/likely GA |  |  |  |  |  |  |  |  |  |  |  |
|  | L536V | known/likely GA |  |  |  |  |  |  |  |  |  |  |  |
|  | Y537S | known/likely GA |  |  |  |  |  |  |  |  |  |  |  |
| 44 | Q414_C417>H | VUS | Unknown | | | | | | | | | ER+/unk | 1.24 |
|  | D538G | known/likely GA |  |  |  |  |  |  |  |  |  |  |  |
| 45 | ESR1-AKAP12 | known/likely GA | Unknown | | | | | | | | | ER+/HER2- | 25.97 |
|  | Y537D | known/likely GA |  |  |  |  |  |  |  |  |  |  |  |
|  | D538G | known/likely GA |  |  |  |  |  |  |  |  |  |  |  |
|  | Y537C | known/likely GA |  |  |  |  |  |  |  |  |  |  |  |
| 46 | D538G | known/likely GA | Yes | Yes | Yes - letrozole | Yes | No | Yes - anastrazole | Yes | capecitabine | response in chest metastasis in last scan but progression in liver metastasis | ER+/HER2- | 0.22 |
| 47 | Y537N | known/likely GA | Yes | No | Yes - exemestane | No | No | Yes - exemestane | Yes | ado-trastuzumab emtansine | progression | ER+/HER2+ | 1.39 |
|  | Y537S | known/likely GA |  |  |  |  |  |  |  |  |  |  |  |
| 48 | D538G | known/likely GA | Unknown | | | | | | | | | ER+/HER2- | 1.92 |
| 49 | E380Q | known/likely GA | Unknown | | | | | | | | | ER+/HER2- | 16.95 |
|  | D538G | known/likely GA |  |  |  |  |  |  |  |  |  |  |  |
|  | Y537S | known/likely GA |  |  |  |  |  |  |  |  |  |  |  |
| 50 | amplification | known/likely GA | No | No | Yes - anastrazole | No | No | No | No | unknown | unknown | ER+/HER2- | 0.18 |
| 51 | H524L | VUS | Unknown | | | | | | | | | ER+/HER2- | 25.54 |
| 52 | D538G | known/likely GA | No | No | Yes - anastrazole, exemestane, letrozole | No | No | Yes - letrozole, exemestane | Yes | fulvestrant | responding | ER+/HER2- | 2.7 |
| 53 | D538G | known/likely GA | received chemotherapy, tamoxifen, anastrazole, exemestane, fulvestrant - not stated if in adjuvant/neoadjuvant or metastatic setting | | | | | | | off-therapy | evidence of metastatic disease | ER+/HER2- | 28.26 |
| 54 | ESR1-NKAIN2 | known/likely GA | Unknown | | | | | | | | | ER+/HER2- | 12.85 |
|  | Y537S | known/likely GA |  |  |  |  |  |  |  |  |  |  |  |
| 55 | V422del | known/likely GA | Metastatic at diagnosis | | | No | No | Yes - anastrazole | Yes | fulvestrant | progression | ER+/HER2- | 6.32 |
|  | Y537S | known/likely GA |  |  |  |  |  |  |  |  |  |  |  |
|  | D538G | known/likely GA |  |  |  |  |  |  |  |  |  |  |  |
| 56 | R352M | VUS | Unknown | | | | | | | | | ER+/HER2- | 11.66 |
| 57 | D538G | known/likely GA | Yes | Yes | No | Yes | No | Yes - letrozole, exemestane, anastrazole | No | off-therapy | progression at last scan | ER+/HER2- | 1.88 |
|  | Y537N | known/likely GA |  |  |  |  |  |  |  |  |  |  |  |
|  | Y537S | known/likely GA |  |  |  |  |  |  |  |  |  |  |  |
| 58 | L536P | known/likely GA | Unknown | | | | | | | | | ER+/HER2+ | 2.03 |
|  | D538G | known/likely GA |  |  |  |  |  |  |  |  |  |  |  |
|  | S463P | known/likely GA |  |  |  |  |  |  |  |  |  |  |  |
| 59 | Y537S | known/likely GA | Yes | Yes | Yes - letrozole | Yes | No | Yes - anastrazole, exemestane | No | letrozole/palbociclib | progression of liver metastasis | ER+/HER2- | 27.33 |
|  | D538G | known/likely GA |  |  |  |  |  |  |  |  |  |  |  |
| 60 | D538G | known/likely GA | Unknown | | | Yes | No | Yes - letrozole | No | unknown | unknown | ER+/HER2- | 2.27 |
| 61 | Y537S | known/likely GA | Unknown | | | | | | | | | ER+/HER2- | 11.76 |
| 62 | E380Q | known/likely GA | Unknown | | | Yes | No | Yes - letrozole | No | Taxol | progression of liver metastasis | ER+/HER2- | 3.03 |
| 63 | D538G | known/likely GA | Unknown | | | | | | | | | ER+/HER2- | 8.08 |
| 64 | amplification | known/likely GA | Unknown | | | Unknown | Unknown | Yes - anastrazole | Unknown | anastrazole | progression | ER+/HER2- | 18.99 |
|  | Y537S | known/likely GA |  |  |  |  |  |  |  |  |  |  |  |
|  | P535T | VUS |  |  |  |  |  |  |  |  |  |  |  |
| 65 | E542K | VUS | Unknown | | | | | | | | | ER+/HER2- | 40.77 |
|  | L539P | VUS |  |  |  |  |  |  |  |  |  |  |  |
|  | H356D | VUS |  |  |  |  |  |  |  |  |  |  |  |
|  | D538G | known/likely GA |  |  |  |  |  |  |  |  |  |  |  |
| 66 | Y537S | known/likely GA | Unknown | | | | | | | | | ER+/unk | 39.62 |
|  | D538G | known/likely GA |  |  |  |  |  |  |  |  |  |  |  |
| 67 | D538G | known/likely GA | Unknown | | | Yes | No | Yes - letrozole | No | pembrolizumab | stable disease | ER+/HER2+ | 61.62 |
| 68 | D538G | known/likely GA | No | No | Yes - anastrazole | Yes | Yes | Yes - exemestane | Yes | fulvestrant/herceptin | progression at last scan | ER+/HER2- | 4.92 |
|  | Y537N | known/likely GA |  |  |  |  |  |  |  |  |  |  |  |
| 69 | Y537N | known/likely GA | Unknown | | | Yes | No | Yes - letrozole, exemestane | Yes | letrozole | unknown | ER+/HER2+ | 4.19 |
|  | Y537S | known/likely GA |  |  |  |  |  |  |  |  |  |  |  |
| 70 | Y537C | known/likely GA | Yes | Yes | No | Yes | Yes | Yes - anastrazole | Yes | tamoxifen/everolimus | stable at last scan | ER+/HER2- | 1.17 |
| 71 | D538G | known/likely GA | Unknown | | | Yes | No | Yes - letrozole, exemestane | Yes | exemestane | progression | ER+/HER2- | 2.29 |
| 72 | D538G | known/likely GA | Unknown | | | | | | | | | ER+/HER2- | 0.48 |
|  | Y537S | known/likely GA |  |  |  |  |  |  |  |  |  |  |  |
| 73 | Y537C | known/likely GA | Yes | Yes | No | No | No | Yes - anastrazole | No | unknown | unknown | ER+/HER2- | 19.56 |
|  | S463P | known/likely GA |  |  |  |  |  |  |  |  |  |  |  |
|  | E380Q | known/likely GA |  |  |  |  |  |  |  |  |  |  |  |
|  | D538G | known/likely GA |  |  |  |  |  |  |  |  |  |  |  |
|  | T347A | VUS |  |  |  |  |  |  |  |  |  |  |  |
|  | M388I | VUS |  |  |  |  |  |  |  |  |  |  |  |
|  | H524L | VUS |  |  |  |  |  |  |  |  |  |  |  |
| 74 | E380Q | known/likely GA | Yes | No | No | Yes | No | Yes - letrozole | Yes | Gemcitabine | stable | ER+/HER2- | 0.21 |
| 75 | Y537S | known/likely GA | Unknown | | | | | | | | | ER+/HER2- | 1.08 |
| 76 | Y537S | known/likely GA | Yes | Yes | No | No | No | Yes - letrozole, exemestane, anastrazole | Yes | fulvestrant/palbociclib | progression | ER+/HER2- | 3.17 |
| 77 | D538G | known/likely GA | Unknown | | | | | | | | | ER+/HER2- | 3.58 |
|  | L536R | known/likely GA |  |  |  |  |  |  |  |  |  |  |  |
| 78 | Y537S | known/likely GA | Unknown | | | | | | | | | ER+/HER2- | 22.58 |
| 79 | D538G | known/likely GA | Unknown | | | | | | | | | ER+/HER2- | 0.76 |
| 80 | D538G | known/likely GA | No | No | Yes - anastrazole | No | No | Yes - letrozole | Yes | fulvestrant/everolimus | unknown | ER+/HER2- | 8.09 |
|  | E380Q | known/likely GA |  |  |  |  |  |  |  |  |  |  |  |
| 81 | D538G | known/likely GA | Unknown | | | | | | | | | ER+/HER2- | 26.48 |
| 82 | E380Q | known/likely GA | Unknown | | | Yes | No | Yes - exemestane, letrozole | Yes | unknown | unknown | ER+/HER2- | 32.58 |
|  | G442R | VUS |  |  |  |  |  |  |  |  |  |  |  |
| 83 | L536P | known/likely GA | received chemotherapy, tamoxifen, letrozole - not stated if in adjuvant/neoadjuvant or metastatic setting; carboplatin/paclitaxel and doxorubicin received in metastatic setting | | | | | | Yes | unknown | unknown | ER+/HER2- | 10.59 |
| 84 | Y537S | known/likely GA | Yes | Yes | No | Yes | No | Yes - letrozole, exemestane | Yes | capecitabine | response in original liver lesion but new lesions apparent | ER+/HER2- | 16.59 |
| 85 | wild type | none | Unknown | | | | | | | | | ER+/HER2- | 57.72 |
| 86 | wild type | none | Unknown | | | | | | | | | ER+/HER2- | 0.28 |
| 87 | wild type | none | Yes | No | Yes | Yes | No | Yes - letrozole, exemestane | Yes | Navelbine | progression at last scan | ER+/HER2- | 0.38 |
| 88 | wild type | none | Unknown | | | | | | | | | ER+/HER2- | 14.37 |
| 89 | wild type | none | Yes | Yes | Yes - anastrazole | Yes | No | Yes - letrozole, exemestane | No | Vinorelbine | unknown | ER+/HER2- | 0.13 |
| 90 | wild type | none | Metastatic at diagnosis | | | Yes | No | Yes - anastrazole, letrozole | No | Gemcitabine/carboplatin | progression at last scan | ER+/HER2- | 35.97 |
| 91 | wild type | none | Unknown | | | | | | | | | ER+/HER2- | 2.98 |
| 92 | wild type | none | No | No | No | Yes | Yes | Yes - anastrazole, exemestane, letrozole | Yes | capecitabine | progression | ER+/HER2- | 2.15 |
| 93 | wild type | none | Unknown | | | | | | | | | ER+/HER2- | 3.6 |
| 94 | wild type | none | Unknown | | | | | | | | | ER+/HER2+ | 3.47 |
| 95 | wild type | none | Unknown | | | | | | | | | ER+/HER2+ | 24.09 |
| 96 | wild type | none | Unknown | | | | | | | | | ER+/HER2- | 0.5 |
| 97 | wild type | none | Yes | Yes | No | Yes | No | Yes - exemestane | Yes | Gemcitabine/cisplatin | progression at last scan | ER+/unk | 0.5 |
| 98 | wild type | none | Unknown | | | | | | | | | ER+/HER2- | 0.58 |
| 99 | wild type | none | Unknown | | | | | | | | | ER+/HER2- | 1.74 |
| 100 | wild type | none | Unknown | | | | | | | | | ER+/HER2- | 0.4 |
| 101 | wild type | none | Metastatic at diagnosis | | | Yes | No | Yes - anastrazole | Yes | unknown | unknown | ER+/HER2- | 3.39 |
| 102 | wild type | none | Unknown | | | | | | | | | ER+/HER2- | 1.55 |
| 103 | wild type | none | Yes | Yes | No | Yes | No | No | Yes | capecitabine | progression of bone metastases | ER+/unk | 0.39 |
| 104 | wild type | none | Unknown | | | | | | | | | ER+/unk | 1.02 |
| 105 | wild type | none | Unknown | | | | | | | | | ER+/HER2- | 29.85 |
| 106 | wild type | none | Unknown | | | | | | | | | ER+/HER2- | 5.11 |
| 107 | wild type | none | Unknown | | | | | | | | | ER+/HER2- | 0.74 |
| 108 | wild type | none | Unknown | | | | | | | | | ER+/unk | 1.24 |
| 109 | wild type | none | Unknown | | | | | | | | | ER+/unk | 0 |
| 110 | wild type | none | Unknown | | | | | | | | | ER+/HER2- | 0 |
| 111 | wild type | none | Yes | Yes | No | Yes | Yes | Yes - exemestane, letrozole | Yes | letrozole/palbociclib | unknown | ER+/HER2- | 0 |
| 112 | wild type | none | Unknown | | | | | | | | | ER+/HER2- | 4.37 |
| 113 | wild type | none | Unknown | | | | | | | | | ER+/HER2- | 59.48 |
| 114 | wild type | none | Unknown | | | | | | | | | ER+/HER2- | 0 |
| 115 | wild type | none | Yes | No | Yes | No | Yes | No | No | Tamoxifen | responding | ER+/unk | 0 |
| 116 | wild type | none | Yes | No | No | Yes | No | Yes - anastrazole, exemestane | Yes | unknown | unknown | ER+/HER2- | 0 |
| 117 | wild type | none | Yes | No | No | Yes | No | Yes - letrozole, exemestane | Yes | exemestane/everolimus | increasing CA27.29 | ER+/HER2- | 0.23 |
| 118 | wild type | none | Yes | Yes | No | No | No | Yes - letrozole | No | ado-trastuzumab emtansine | stable | ER+/HER2+ | 0.2 |
| 119 | wild type | none | Unknown | | | | | | | | | ER+/HER2- | 0.16 |
| 120 | wild type | none | Unknown | | | | | | | | | ER+/HER2- | 4.29 |
| 121 | wild type | none | Unknown | | | | | | | | | ER+/unk | 0.13 |
| 122 | wild type | none | No | No | No | Yes | No | Yes - anastrazole | No | paclitaxel | progression | ER+/HER2- | 0.73 |
| 123 | wild type | none | Metastatic at diagnosis | | | No | No | Yes - letrozole | Yes | fulvestrant/palbociclib | progression | ER+/HER2- | 0 |
| 124 | wild type | none | No | Yes | No | Yes | No | Yes - anastrazole | No | trastuzumab/pertuzumab | unknown | ER+/HER2+ | 5.4 |
| 125 | wild type | none | Unknown | | | | | | | | | ER+/HER2+ | 1.9 |
| 126 | wild type | none | No | No | No | not applicable: Stage 1B | | | | unknown | unknown | ER+/HER2- | 0 |
| 127 | wild type | none | Metastatic at diagnosis | | | Yes | No | Yes - letrozole, exemestane | Yes | exemestane/everolimus | progression | ER+/HER2- | 20.49 |
| 128 | wild type | none | Yes | Yes | Yes - anastrazole | No | No | Yes - anastrazole, exemestane, letrozole | Yes | letrozole/everolimus | unknown | ER+/HER2- | 8.98 |
| 129 | wild type | none | Unknown | | | | | | | | | ER+/HER2- | 0.25 |
| 130 | wild type | none | Metastatic at diagnosis | | | No | No | Yes - letrozole | No | unknown | unknown | ER+/HER2- | 9.72 |
| 131 | wild type | none | Metastatic at diagnosis | | | Yes | No | No | Yes | trastuzumab/fulvestrant | increasing CA27.29 and CEA | ER+/HER2+ | 0.32 |
| 132 | wild type | none | Yes | Yes | No | No | No | Yes - letrozole | No | clinical trial | NED | ER+/HER2- | 0 |
| 133 | wild type | none | Unknown | | | | | | | | | ER+/HER2- | 0 |
| 134 | wild type | none | Metastatic at diagnosis | | | Yes | No | Yes - anastrazole | No | everolimus/AI | unknown | ER+/HER2- | 0.19 |
| 135 | wild type | none | Yes | Yes | No | No | Yes | Yes - exemestane, letrozole | Yes | capecitabine | decreasing CA27.29 | ER+/HER2- | 0.26 |
| 136 | wild type | none | Unknown | | | | | | | | | ER+/unk | 0.99 |
| 137 | wild type | none | Unknown | | | | | | | | | ER+/HER2- | 3.03 |
| 138 | wild type | none | Unknown | | | Yes | Unknown | | | | | ER+/HER2- | 0.52 |
| 139 | wild type | none | Yes | Yes | Yes - letrozole | Yes | No | Yes - anastrazole, exemestane | Yes | lapatinib/capecitabine | stable at last scan, increasing CA27.29 | ER+/HER2+ | 14.96 |
| 140 | wild type | none | Unknown | | | | | | | | | ER+/unk | 4.29 |
| 141 | wild type | none | Unknown | | | | | | | | | ER+/HER2- | 0.72 |
| 142 | wild type | none | Unknown | | | | | | | | | ER+/HER2- | 0.63 |
| 143 | wild type | none | Unknown | | | Yes | No | Yes - exemestane | No | trastuzumab/navelbine | unknown | ER+/HER2+ | 3.09 |
| 144 | wild type | none | Unknown | | | | | | | | | ER+/HER2- | 0 |
| 145 | wild type | none | Unknown | | | | | | | | | ER+/HER2- | 0 |
| 146 | wild type | none | Yes | No | Yes - anastrazole | not applicable: Stage 3C | | | | anastrazole | unknown | ER+/HER2- | 1.47 |
| 147 | wild type | none | No | No | No | No | No | No | No | No | unknown | ER+/HER2- | 15 |
| 148 | wild type | none | Yes | No | Yes - anastrazole, exemestane | Yes | No | No | Yes | eribulin | progression at last scan | ER+/HER2- | 0.57 |
| 149 | wild type | none | Unknown | | | | | | | | | ER+/HER2- | 1.74 |
| 150 | wild type | none | Metastatic at diagnosis | | | Yes | No | Yes | Yes | abraxane | evidence of metastatic disease | ER+/HER2- | 0.46 |
| 151 | wild type | none | Unknown | | | | | | | | | ER+/HER2- | 21.58 |
| 152 | wild type | none | Yes | No | No | Yes | No | Yes - exemestane | Yes | radiation | progression | ER+/HER2- | 0.25 |
| 153 | wild type | none | Unknown | | | | | | | | | ER+/unk | 0 |
| 154 | wild type | none | Unknown | | | | | | | | | ER+/HER2- | 0 |
| 155 | wild type | none | Yes | No | Yes - letrozole | Yes | Yes | Yes - anastrazole, letrozole, exemestane | Yes | ado-trastuzumab emtansine | progression | ER+/HER2+ | 2.7 |
| 156 | wild type | none | Yes | No | Yes - anastrazole | Yes | No | Yes - exemestane | Yes | eribulin | unknown | ER+/HER2- | 20.6 |
| 157 | wild type | none | Yes | No | Yes - letrozole | Yes | No | Yes - exemestane, letrozole | Yes | taxol | unknown | ER+/HER2- | 0 |
| 158 | wild type | none | Yes | Yes | No | Yes | No | No | Yes | eribulin | stable | ER+/HER2- | 0 |
| 159 | wild type | none | Unknown | | | | | | | | | ER+/unk | 0.38 |
| 160 | wild type | none | Unknown | | | | | | | | | ER+/HER2- | 0.21 |
| 161 | wild type | none | Unknown | | | | | | | | | ER+/HER2- | 0.26 |
| 162 | wild type | none | Unknown | | | | | | | | | ER+/HER2- | 0.23 |
| 163 | wild type | none | No | No | No | Yes | Yes | Yes - letrozole | No | capecitabine | progression at last scan | ER+/HER2- | 0 |
| 164 | wild type | none | Unknown | | | | | | | | | ER+/HER2+ | 0.3 |
| 165 | wild type | none | Metastatic at diagnosis | | | Yes | No | Yes - anastrazole, exemestane, | No | unknown | increasing CA27.29 | ER+/HER2- | 9.27 |
| 166 | wild type | none | Unknown | | | | | | | | | ER+/HER2- | 0 |
| 167 | wild type | none | Yes | No | Yes - anastrazole | Yes | No | No | Yes | Capecitabine | progression | ER+/HER2- | 0.28 |
| 168 | wild type | none | Unknown | | | | | | | | | ER+/unk | 19.91 |
| 169 | wild type | none | Metastatic at diagnosis | | | Yes | No | Yes - anastrazole, letrozole, exemestane | Yes | docetaxel | progression | ER+/HER2- | 1.38 |
| 170 | wild type | none | Yes | Yes | No | No | No | No | No | unknown | evidence of metastatic disease | ER+/unk | 0.33 |
| 171 | wild type | none | Unknown | | | | | | | | | ER+/HER2+ | 13.92 |
| 172 | wild type | none | Yes | Yes | No | Yes | No | Yes - anastrazole, exemestane, letrozole | Yes | capecitabine | progression | ER+/HER2- | 7.84 |
| 173 | wild type | none | Unknown | | | Yes | Unknown | | | | | ER+/HER2- | 0.23 |
| 174 | wild type | none | Unknown | | | not applicable: stage 2A | | | | unknown | unknown | ER+/HER2- | 0.88 |
| 175 | wild type | none | Yes | Unknown | Unknown | Yes | Unknown | Unknown | Yes | capecitabine | unknown | ER+/unk | 9.29 |
| 176 | wild type | none | Unknown | | | | | | | | | ER+/HER2- | 0.72 |
| 177 | wild type | none | Unknown | | | | | | | | | ER+/HER2- | 0 |
| 178 | wild type | none | Yes | Yes | No | Yes | No | Yes - letrozole, exemestane | Yes | cyclophosphamide | progression | ER+/unk | 0.49 |
| 179 | wild type | none | Yes | No | Yes | Yes | No | No | No | capecitabine | progression | ER+/HER2- | 0.32 |
| 180 | wild type | none | Unknown | | | | | | | | | ER+/HER2- | 7.36 |
| 181 | wild type | none | Unknown | | | | | | | | | ER+/unk | 0.29 |
| 182 | wild type | none | Yes | No | No | Yes | No | Yes - exemestane | Yes | fulvestrant/palbociclib | unknown | ER+/HER2- | 3.16 |
| 183 | wild type | none | Unknown | | | | | | | | | ER+/unk | 0 |
| 184 | wild type | none | No | No | No | not applicable: stage 2 post-mastectomy | | | | | | ER+/HER2+ | 2.55 |
| 185 | wild type | none | Yes | Yes | Yes - letrozole | not applicable: stage 2 recurrence | | | | Letrozole | Recurrent | ER+/HER2- | 0 |
| 186 | wild type | none | Yes | No | Yes - anastrazole | Yes | No | Yes - letrozole | No | capecitabine | unknown | ER+/HER2- | 0 |
| 187 | wild type | none | Yes | Yes | No | Yes | No | Yes - anastrazole | Yes | taxol | evidence of metastatic disease - mixed response | ER+/HER2- | 4.72 |
| 188 | wild type | none | Yes | Yes | Yes - anastrazole, exemestane | Yes | No | Yes - exemestane | No | unknown | unknown | ER+/HER2+ | 0.69 |
| 189 | wild type | none | Unknown | | | | | | | | | ER+/unk | 19.19 |
| 190 | wild type | none | Yes | No | Yes - anastrazole | Yes | No | No | Yes | taxol | unknown | ER+/HER2- | 1.64 |
| 191 | wild type | none | Unknown | | | | | | | | | ER+/HER2- | 17.06 |
| 192 | wild type | none | Unknown | | | | | | | | | ER+/HER2- | 0 |
| 193 | wild type | none | Yes | No | No | No | No | No | No | unknown | unknown | ER+/HER2- | 7.13 |
| 194 | wild type | none | received aromatase inhibitor - unknown if in adjuvant/neoadjuvant or metastatic setting | | | | | | | | | ER+/HER2- | 73.61 |
| 195 | wild type | none | Unknown | | | | | | | | | ER+/unk | 0 |
| 196 | wild type | none | Unknown | | | | | | | | | ER+/HER2- | 1.48 |
| 197 | wild type | none | Yes | No | No | Yes | No | Yes - letrozole | Yes | exemestane/everolimus | stable at last scan | ER+/HER2- | 0.28 |
| 198 | wild type | none | Metastatic at diagnosis | | | Yes | No | Yes - letrozole | No | letrozole/everolimus | no evidence of metastatic disease at last scan | ER+/HER2- | 1.49 |
| 199 | wild type | none | Yes | Yes | Yes - anastrazole | No | No | No | No | tamoxifen | progression to metastatic disease | ER+/HER2- | 2.06 |
| 200 | wild type | none | Unknown | | | | | | | | | ER+/HER2- | 4.52 |
| 201 | wild type | none | Unknown | | | | | | | | | ER+/HER2- | 13.15 |
| 202 | wild type | none | Unknown | | | Yes | Unknown | | | | | ER+/HER2- | 2.41 |
| 203 | wild type | none | Yes | Yes | Yes - anastrazole, exemestane | Yes | No | Yes - letrozole | No | letrozole/capecitabine | evidence of metastatic disease, increasing CA27.29 | ER+/HER2- | 1.08 |
| 204 | wild type | none | Yes | No | No | Yes | No | Yes - letrozole | No | letrozole/everolimus | no evidence of metastatic disease at last scan | ER+/HER2- | 0.37 |
| 205 | wild type | none | Yes | Yes | Yes - letrozole | not applicable: Stage 3 | | | | unknown | no evidence of metastatic disease at last scan | ER+/HER2- | 0.59 |
| 206 | wild type | none | Yes | No | Yes - letrozole | Yes | No | Yes - letrozole, exemestane | No | navelbine | increasing CA27.29 | ER+/HER2- | 64.23 |
| 207 | wild type | none | Unknown | | | | | | | | | ER+/HER2- | 20.85 |
| 208 | wild type | none | Unknown | | | | | | | | | ER+/HER2- | 0 |
| 209 | wild type | none | Unknown | | | | | | | | | ER+/HER2- | 0 |
| 210 | wild type | none | Yes | Yes | No | New metastatic diagnosis at sample | | | | | | ER+/HER2- | 1.27 |
| 211 | wild type | none | Yes | Yes | No | Yes | No | Yes - letrozole | No | paclitaxel | progression - new metastatic mass | ER+/HER2- | 0 |
| 212 | wild type | none | Unknown | | | | | | | | | ER+/HER2- | 2.93 |
| 213 | wild type | none | Yes | Yes | No | Yes | No | Yes - letrozole, exemestane | Yes | unknown | unknown | ER+/HER2- | 0 |
| 214 | wild type | none | Metastatic at diagnosis | | | Yes | No | No | Yes | fulvestrant/bicalutamide | responding | ER+/HER2- | 0.14 |
| 215 | wild type | none | Unknown | | | | | | | | | ER+/HER2- | 0.88 |
| 216 | wild type | none | Unknown | | | | | | | | | ER+/HER2- | 2.58 |
| 217 | wild type | none | Unknown | | | | | | | | | ER+/HER2- | 0 |
| 218 | wild type | none | Unknown | | | | | | | | | ER+/HER2- | 0 |
| 219 | wild type | none | Yes | Yes | Yes - anastrazole | not applicable: stage 2 | | | | unknown | unknown | ER+/HER2- | 0 |
| 220 | wild type | none | No | Yes | No | Yes | No | Yes - exemestane | No | unknown | unknown | ER+/HER2- | 1.75 |
| 221 | wild type | none | Yes | Yes | No | Yes | No | No | No | trastuzumab/cisplatin/olaparib | responding | ER+/HER2+ | 0.7 |
| 222 | wild type | none | Yes | Toremifene | Yes - letrozole | Unknown | | | | | | ER+/unk | 0.21 |
| 223 | wild type | none | No | No | No | Yes | No | Yes - anastrazole | No | anastrazole | stable disease | ER+/HER2- | 3.76 |
| 224 | wild type | none | Unknown | | | | | | | | | ER+/unk | 4.48 |
| 225 | wild type | none | Yes | No | Yes - exemestane | Yes | No | No | Yes | fulvestrant | NED | ER+/HER2- | 0.3 |
| 226 | wild type | none | Yes | No | No | Yes | Raloxifene | No | No | cisplatin/5-FU/capecitabine/everolimus | response - NED | ER+/HER2- | 0.46 |
| 227 | wild type | none | Yes | No | Yes | No | No | Yes - letrozole, exemestane | Yes | fulvestrant/palbociclib | progression | ER+/HER2- | 3.97 |
| 228 | wild type | none | Unknown | | | | | | | | | ER+/HER2- | 26 |
| 229 | wild type | none | Unknown | | | | | | | | | ER+/HER2- | 11.15 |
| 230 | wild type | none | Unknown | | | | | | | | | ER+/HER2- | 8.23 |
| 231 | wild type | none | received chemotherapy, letrozole - not stated if in adjuvant/neoadjuvant or metastatic setting | | | | | | | unknown | unknown | ER+/HER2- | 0 |
| 232 | wild type | none | No | No | Yes - letrozole, exemestane | Yes | No | No | No | trastuzumab/pertuzumab | unknown | ER+/HER2+ | 0.15 |
| 233 | wild type | none | yes | Unknown | | | | | | | | ER+/HER2- | 0.2 |
| 234 | wild type | none | yes | Unknown | | | | | | | | ER+/HER2- | 0 |
| 235 | wild type | none | Yes | Yes | Yes - letrozole | No | No | Yes - letrozole | No | letrozole/everolimus/bicalutamide | stable disease | ER+/HER2- | 0 |
| 236 | wild type | none | Yes | Yes | Yes - anastrazole, letrozole | Yes | Yes | Yes - exemestane | Yes | gemcitabine | evidence of metastatic disease | ER+/HER2- | 39.55 |
| 237 | wild type | none | No | No | Yes - letrozole | not applicable: stage 1 | | | | adjuvant letrozole | NED | ER+/HER2- | 0.16 |
| 238 | wild type | none | No | No | No | New metastatic diagnosis at sample | | | | | | ER+/HER2- | 0 |
| 239 | wild type | none | Unknown | | | | | | | | | ER+/HER2- | 0.94 |
| 240 | wild type | none | No | Yes | Yes - letrozole | Yes | No | Yes - letrozole | Yes | fulvestrant/palbociclib | progression | ER+/HER2- | 0.26 |
| 241 | wild type | none | No | Yes | Yes - letrozole | Unknown | | | | | | ER+/HER2+ | 0.13 |
| 242 | wild type | none | Unknown | | | | | | | | | ER+/HER2- | 3.73 |
| 243 | wild type | none | Yes | Yes | No | Yes | No | Yes - anastrazole, letrozole, exemestane | Yes | eribulin | stable disease | ER+/HER2- | 0.48 |
| 244 | wild type | none | No | No | No | Yes | No | No | No | trastuzumab | responding at last scan | ER+/HER2+ | 3.39 |
| 245 | wild type | none | Yes | No | Yes - anastrazole, letrozole | not applicable: stage 3 | | | | unknown | unknown | ER+/HER2- | 0 |
| 246 | wild type | none | Unknown | | | | | | | | | ER+/HER2+ | 0.2 |
| 247 | wild type | none | Yes | Yes | No | No | No | Yes - anastrazole, exemestane | No | ado-trastuzumab emtansine | unknown | ER+/HER2+ | 4.79 |
| 248 | wild type | none | Unknown | | | Yes | No | Yes - letrozole | No | unknown | unknown | ER+/HER2+ | 0 |
| 249 | wild type | none | Unknown | | | No | No | Yes - letrozole | No | letrozole/palbociclib | unknown | ER+/HER2- | 0 |
| 250 | wild type | none | Yes | Yes | Yes - letrozole | Yes | Yes | No | No | unknown | unknown | ER+/HER2- | 31.69 |
| 251 | wild type | none | Unknown | | | No | No | Yes - letrozole | No | unknown | unknown | ER+/HER2- | 0.34 |
| 252 | wild type | none | Unknown | | | | | | | | | ER+/unk | 0 |
| 253 | wild type | none | Yes | No | No | No | No | No | No | unknown | unknown | ER+/HER2+ | 8.03 |
| 254 | wild type | none | Unknown | | | | | | | | | ER+/HER2- | 0 |
