# Supplementary material for: Hybrid capture-based genomic profiling of circulating tumor DNA from patients with estrogen receptor-positive metastatic breast cancer
Source: Ann Oncol. 2017 Aug 31;28(11):2866–73. doi: 10.1093/annonc/mdx490 (PMC5834148; doi:10.1093/annonc/mdx490)
Supplement: mdx490_supplementary_methods_corrected [file mdx490_supplementary_methods_corrected.docx]

**Supplementary Methods**

Approval for this study, including a waiver of informed consent and HIPAA waiver of authorization, was obtained from the Western Institutional Review Board (Protocol 20152817). Peripheral blood samples from 254 patients with ER+ BC were submitted by clinicians as part of routine clinical care (5/2016-3/2017); blood samples from 74 patients with ER-negative breast cancer were used for comparison of *ESR1* GAs. ER (IHC), HER2 (IHC/FISH), stage and treatment were determined by review of available medical records (Supplementary Table S2).

Genomic profiling of ctDNA was performed using the FoundationACT ctDNA assay in a CLIA-certified, CAP-accredited laboratory (Foundation Medicine [FM]). Plasma was isolated from 20 mL of whole blood. ≥20 ng cell-free DNA was extracted to create adapted sequencing libraries before hybrid capture and sample-multiplexed sequencing (Illumina HiSeq 2500 or 4000) to a median unique coverage depth of 7503X for 62 genes (Supplementary Table S1). Allele frequency (AF) represents the percentage of mutant DNA allele reads relative to total DNA allele reads. Results were analyzed for substitutions (AF≥0.1%), short insertions/deletions (AF≥0.1%), rearrangements, and copy number amplification [1]. Custom filtering was applied to report GAs and remove benign germline events as described [2]. Maximum somatic allele frequency (MSAF) measures the AF of all somatic alterations per sample; the maximum AF measured is defined as MSAF, which provides an estimate of the ctDNA fraction in plasma.

Temporally matched tissue samples were sequenced as described [2, 3]. For paired ctDNA-tissue samples, only GAs that were analyzed in both tissue and ctDNA were compared. Genomics datasets of tissue for comparison were from the FM database (11,488 BC samples, 851 confirmed ER+) [2], or published studies [4–6]. Data from [4, 5] were extracted from cBioPortal in March 2017 [7].

1. Stephens, P. J., Clark, T., Kennedy, M., He, J., Young, G., Zhao, M., … Ross, J. S. (2016). Analytic validation of a clinical circulating tumor DNA assay for patients with solid tumors. *Annals of Oncology*, *27*(suppl_6). doi:10.1093/annonc/mdw380.01

2. Hartmaier, R. J., Albacker, L., Chmielecki, J., Bailey, M., He, J., Goldberg, M. E., … Lipson, D. (2017). High-throughput genomic profiling of adult solid tumors reveals novel insights into cancer pathogenesis. *Cancer research*, canres.2479.2016. doi:10.1158/0008-5472.CAN-16-2479

3. Frampton, G. M., Fichtenholtz, A., Otto, G. A., Wang, K., Downing, S. R., He, J., … Yelensky, R. (2013). Development and validation of a clinical cancer genomic profiling test based on massively parallel DNA sequencing. *Nature biotechnology*, *31*(11), 1023–31. doi:10.1038/nbt.2696

4. Ciriello, G., Gatza, M. L., Beck, A. H., Wilkerson, M. D., Rhie, S. K., Pastore, A., … Perou, C. M. (2015). Comprehensive Molecular Portraits of Invasive Lobular Breast Cancer. *Cell*, *163*(2), 506–19. doi:10.1016/j.cell.2015.09.033

5. Lefebvre, C., Bachelot, T., Filleron, T., Pedrero, M., Campone, M., Soria, J.-C., … André, F. (2016). Mutational Profile of Metastatic Breast Cancers: A Retrospective Analysis. *PLOS Medicine*, *13*(12), e1002201. doi:10.1371/journal.pmed.1002201

6. Fumagalli, D., Wilson, T. R., Salgado, R., Lu, X., Yu, J., O’Brien, C., … Loi, S. (2016). Somatic mutation, copy number and transcriptomic profiles of primary and matched metastatic estrogen receptor-positive breast cancers. *Annals of Oncology*, *27*(10), 1860–1866. doi:10.1093/annonc/mdw286

7. Cerami, E., Gao, J., Dogrusoz, U., Gross, B. E., Sumer, S. O., Aksoy, B. A., … Schultz, N. (2012). The cBio Cancer Genomics Portal: An Open Platform for Exploring Multidimensional Cancer Genomics Data: Figure 1. *Cancer Discovery*, *2*(5), 401–404. doi:10.1158/2159-8290.CD-12-0095
